# Drying Rate and Product Quality Evaluation of Roselle (*Hibiscus sabdariffa *L.) Calyces Extract Dried with Foaming Agent under Different Temperatures

**DOI:** 10.1155/2018/9243549

**Published:** 2018-03-20

**Authors:** Mohamad Djaeni, Andri Cahyo Kumoro, Setia Budi Sasongko, Febiani Dwi Utari

**Affiliations:** Department of Chemical Engineering, Faculty of Engineering, Diponegoro University, Jl. Prof. H. Soedarto, SH, Tembalang, Semarang 50275, Indonesia

## Abstract

The utilisation of roselle (*Hibiscus sabdariffa *L.) calyx as a source of anthocyanins has been explored through intensive investigations. Due to its perishable property, the transformation of roselle calyces into dried extract without reducing their quality is highly challenging. The aim of this work was to study the effect of air temperatures and relative humidity on the kinetics and product quality during drying of roselle extract foamed with ovalbumin and glycerol monostearate (GMS). The results showed that foam mat drying increased the drying rate significantly and retained the antioxidant activity and colour of roselle calyces extract. Shorter drying time was achieved when higher air temperature and/or lower relative humidity was used. Foam mat drying produced dried brilliant red roselle calyces extract with better antioxidant activity and colour qualities when compared with nonfoam mat drying. The results showed the potential for retaining the roselle calyces extract quality under suggested drying conditions.

## 1. Introduction

Roselle (*Hibiscus sabdariffa* L.) is commercially cultivated in some countries like India, Indonesia, Malaysia, Sudan, Egypt, and Mexico [1]. The roselle calyx is brilliant red in colour due to the existence of anthocyanins, such as cyanidin-3-sambubioside, cyanidin-3-glucoside, and delphinidin-3-glucoside [2]. The calyx is usually used to prepare jam, jelly, cakes, ice cream, preserves, and herbal beverage [3]. The consumption of roselle calyx tea has been reported to promote health benefits, which mainly functions as an antioxidant [4]. The relationship between antioxidant activity and anthocyanin of roselle calyx has been reported in the literatures [5, 6]. The total anthocyanin content of roselle calyces is 2.52 g/100 g expressed as delphinidin-3-glucoside [7]. Besides, the roselle extracts also contain ascorbic acid. Wang et al. observed that anthocyanins have many times more antioxidant activity than ascorbic acid [8]. Therefore, the antioxidant activity of roselle extract is predominantly contributed by anthocyanins. However, the use of anthocyanins in food products experienced problems related to their instability during processing and storage caused by direct exposure to heat, oxygen, and light [9]. Mazza and Miniati observed that thermal degradation of anthocyanin of roselle extract was fast at temperatures above 100°C [10].

Microencapsulation technique is one of the methods to maintain anthocyanins stability by entrapping them inside a coating material to reduce direct interactions with external factors, such as temperature, light, moisture, and oxygen. Although spray drying and freeze drying are the most common microencapsulation methods applied in the food and pharmaceutical industries, freeze drying is 30 to 50 times more costly than that of spray drying [11]. However, extreme moisture loss during spray drying may trigger shrinking and deformation of dried particles [12].

Foam mat drying is carried out by transforming liquid and semisolid materials into stable foam by incorporation of air and a foaming agent. It is a good option to shorten drying time and to retain product quality [13]. Ovalbumin is usually chosen as a foaming agent due to the ability of its proteins to generate a dense film around the air bubbles, reducing the surface tension instability and retaining the entrapped air [14]. In addition, glycerol monostearate (GMS) and methyl cellulose (MC) are the other common foaming agents [13, 15]. With the agents, the surface area of product becomes greater for mass and heat transfer. These conditions allow higher drying rates [13].

The foam mat drying can be a potential option for roselle extract drying. However, the application cannot be straightforward. As far as literature survey is being done, no studies have been carried out to investigate the degradation of the anthocyanins, colour, and antioxidant activity as a function of temperature, relative humidity, and time during foam mat drying of roselle extract. This study aimed to study the effect of air temperatures and relative humidity on the kinetics and product quality during foam drying of roselle extract. As indicators, the drying time and the quality of roselle extract in terms of antioxidant activity and colour were evaluated. The results were also compared with conventional convective drying without foaming agent.

## 2. Materials and Methods

### 2.1. Plant Materials and Chemicals

Traditionally dried brilliant red roselle (*Hibiscus sabdariffa* Linn.) calyces, with average size and with no bruises, were obtained from an herbal medicine market in Solo, Indonesia. 2,2-Diphenyl-1-picrylhydrazyl (DPPH) and ethanol were of analytical grade and purchased from Merck (Darmstadt, Germany).

### 2.2. Roselle Extract Preparation

The calyces were ground using a blender (Panasonic MX-895 M, Japan) and sieved carefully to obtain powder with 0.25 mm in size. The roselle was extracted using water as solvent as previously suggested by Chumsri et al. [16]. One hundred grams of roselle calyces powder was mixed with water in a beaker glass at a mass to volume ratio (gmL^−1^) of 1 : 10 at 50°C under continuous agitation for 1 hour. The extract was then filtered through a nylon filter to obtain brilliant red colour liquid extract.

### 2.3. Foam Drying Process

**Figure 1:**
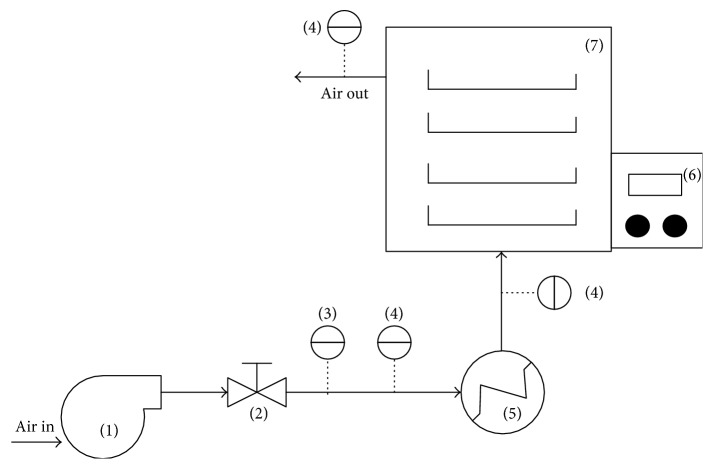
The schematic overview of the tray drying equipment ((1) blower; (2) valve; (3) temperature and relative humidity sensor (T-RH); (4) temperature and velocity sensor (T-V); (5) heater; (6) thermocontroller; (7) drying chamber).

Foam drying was used to reduce the moisture content of the roselle extract [13]. The drying system is depicted in Figure 1. The roselle extract (100 ml) was mixed with 4.23 grams of ovalbumin as the foaming agent and 0.423 grams of GMS as foam stabiliser. After complete mixing, the solution was whipped in a domestic mixer (360 W power) at maximum speed for 20 min to provide the mechanical incorporation of air (the density of foam was 17.73 kg/m^3^). After whipping, the foam was placed on aluminium beds (length 20 cm, width 15 cm and thickness of 0.2 cm). The beds were then put in the dryer which used ambient air as drying medium. Before entering the dryer, the ambient air with relative humidity of 80%, temperature range of 30–32°C, and superficial velocity of 0.22 m s^−1^ was heated up to certain inlet drying temperature (40°C, 50°C, and 60°C) using an electric heater equipped with thermocontroller. So, the relative humidity of ambient air decreased corresponding to the inlet drying temperatures. Because of water releases during drying process, the foam thickness is usually reduced to about 5% of its initial condition. The moisture loss was determined gravimetrically by weighing the samples every 10 min on an electronic scale. In addition, antioxidant activity and colour of the dried extract were also determined accordingly. The experiments were terminated after 120-minute drying times. As a comparison, the roselle extract was also dried without addition of foaming agent and stabiliser (control sample).

### 2.4. Determination of Moisture Content

The moisture content in roselle extract-ovalbumin-glycerol monostearate mixture was measured referring to AOAC method [17]. Briefly, 1 gram of dried extract sample was heated at the temperature of 105°C in the oven until a constant weight was obtained. In this case, the initial moisture content in the extract before drying was 95% (wet basis) or 19 grams of moisture per gram of dry extract (dry basis).

### 2.5. Modelling of Foam Drying

Referring to thin layer drying model, the correlation between moisture ratios with time can be represented by the following [18]:

(1) $$\begin{array}{c} MR=\frac{\left(M_{t}-M_{e}\right)}{\left(M_{0}-M_{e}\right)}, \end{array}$$

where *M*_*t*_ was the moisture content at time *t*, *M*_0_ was the initial moisture content, and *M*_*e*_ was the equilibrium moisture content of the foam, all of them in dry basis (g g^−1^). The equilibrium moisture content in roselle extract with the foaming agent was estimated by considering the composition of the mixture and both equilibrium moistures in roselle and ovalbumin as expressed by [19]

(2) $$\begin{array}{c} M_{e}=\sum x_{i}M_{ei}, \end{array}$$

where *x* was the mass fraction of roselle and ovalbumin, *i* referred to components (roselle and ovalbumin), and *M*_*e*_ was the equilibrium moisture content (g water per g dry solid). The equilibrium moisture of roselle and ovalbumin was influenced by relative humidity or water activity (Aw) and temperature [19, 20]. Langová et al. suggested that the equilibrium moisture of roselle can be extended as follows [19]:

(3) $$\begin{array}{c} {M_{e}=A}^{Aw-B}+C,\\ A=1568.6e^{-0.072T},\\ B=-0.0076T+0.4687,\\ C=-0.1898T+13.49, \end{array}$$

where *A*, *B*, and *C* were the constant in equilibrium moisture content equation and *T* was the drying temperatures (40°C, 50°C, and 60°C). Using the same principles, Christ et al. suggested that the equilibrium moisture of ovalbumin can be satisfactorily predicted using [20]

(4) $$\begin{array}{c} M_{e}={exp}^{\left\{-0.2387-\left(0.0035\left(T+273\right)\right)+\left[1.5933\exp\left(A_{w}\right)\right]\right\}}. \end{array}$$

The drying rate (DR) was estimated based on the moisture ratio (dimensionless moisture) reduction as a function of time as suggested by Franco et al. [18].

(5) $$\begin{array}{c} DR=\frac{\left(M_{t+dt}-M_{t}\right)}{dt}, \end{array}$$

where *M*_*t*+*dt*_ and *M*_*t*_ were the moisture contents at times *t* + *dt* and *t*, respectively. Based on literature surveys, the thin layer model (Newton model) accuracy has been proven to predict the rate of foam drying process [21, 22]. According to Newton model, the drying time for rosella extract can be estimated by

(6) $$\begin{array}{c} t=-\frac{\ln\left(MR\right)}{k_{d}}. \end{array}$$

Here, *k*_*d*_ was the drying rate constant (minute^−1^). The dependency of *k*_*d*_ on temperature (*T*) can be correlated using Arrhenius like equation as shown by

(7) $$\begin{array}{c} k_{d}=k_{0}{exp}^{-E_{a}/R\left(T+273\right)}, \end{array}$$

where *k*_0_ was the preexponential factor (minute^−1^), *E*_*a*_ was the activation energy (J mol^−1^), and *R* was the gas constant (8.32 J mol^−1^ K^−1^).

The mass diffusion coefficient can be calculated using the analytical solution for a flat plate, which was previously used by Franco et al. [18].

(8) $$\begin{array}{c} MR=\left(\;\frac{8}{\pi^{2}}\;\right){exp}^{\left(-D_{if}\pi^{2}t\right)/\tau^{2}}, \end{array}$$

where *D*_if_ was the mass diffusion coefficient (m^2 ^min^−1^), *t* was the drying time (minute), and *τ* was the thickness of the foam (m).

### 2.6. Mass and Heat Transfer Analysis

The mass and heat convective transfer coefficients, *h* and *h*_*m*_, were determined by (9) and (10) using dimensionless numbers obtained from (11) to (14) which were previously used by Franco et al. [18].

(9) h=Nu kod,

(10) hm=Sh DWAd,

(11) Re=ρovoμo,

(12) Nu=0.664 Re1/2Pr1/3,

(13) Sc=μoρoDWA,

(14) Sh=0.664 Re1/2Sc1/3,

(15) DWA=1.87×10−10T+2732.072P,

where Re, Nu, Sc, and Sh were the Reynold, Nusselt, Schmidt, and Sherwood number, respectively, whereas *k*_*o*_, *P*, *T*, *ρ*_*o*_, *v*_*o*_, and *μ*_*o*_ were, respectively, the thermal conductivity (Wm^−1^K^−1^), pressure (atm), temperature (°C), density (kgm^−3^), velocity (ms^−1^), and viscosity (Pa s) of air. In addition, *d* was the material length (m) and *D*_WA_ was the diffusivity of water in air (m^2^s^−1^).

### 2.7. DPPH Free Radical-Scavenging Activity Assay

The capacity of roselle extracts to inhibit the sequestration activity of 2,2-diphenyl-1-picrylhydrazyl (DPPH) free radical was observed according to the method of Brand-Williams et al. [23]. Precisely, 100 *μ*L of ethanolic extract solutions was added to 1.4 mL DPPH radical ethanolic solution (10^−4^ mol L^−1^) in a test tube. The content of the test tube was shaken vigorously and let to react at room temperature for 30 minutes in the dark. The absorbance at 517 nm was then measured against a blank (100 *μ*L of ethanol in 1.4 mL of DPPH radical solution) using a spectrophotometer. The antioxidant activity was calculated as percentage inhibition (%) of DPPH radical formation, as follows:

(16) $$\begin{array}{c} I\left(\;\%\;\right)=\left[\;\frac{A_{0}-A_{s}}{A_{s}}\;\right]100\%, \end{array}$$

where *A*_0_ was absorbance of control blank and *A*_*s*_ was absorbance of sample extract. All determinations were conducted in triplicate.

### 2.8. Colour Measurement

The colour changes of dried roselle extract were observed by measuring *L*, *a*, and *b* values using a Chroma Meter (CR-300, Minolta Co., Ltd., Osaka, Japan) as previously used by Kumar et al. [6]. The total colour degradation during drying process was evaluated based on the changes of total colour ratio (TCR) as suggested by Ahmed et al. [24] and the value was calculated using

(17) $$\begin{array}{c} TCR=\frac{L_{s}a_{s}b_{s}}{L_{o}a_{o}b_{o}}, \end{array}$$

where *L*_*s*_, *a*_*s*_, and *b*_*s*_ were the measured on a sample at a given time, while *L*_*o*_, *a*_*o*_, and *b*_*o*_ were measured on raw roselle extract (before the drying process). All determinations were conducted in triplicate.

### 2.9. Total Anthocyanin Content Measurement

The total anthocyanin content was observed with the method referring to Anuar et al. [25]. The samples (fresh and dried roselle extract) were diluted in 0.025 M KCl buffer (pH 1) and 0.4 M CH3COONa (pH 4.5). The absorbance of the solution was measured at 520 nm and 700 nm using UV-Vis Spectrophotometer (UVmini-1240, Shimadzu, Japan). The total anthocyanin content (TAC), as cyanidin-3-glucoside was calculated using the following [25]:

(18) $$\begin{array}{c} TAC\left(\;mg/L\;\right)=\frac{A\times MW\times DF\times 1000}{\varepsilon \times d}, \end{array}$$

where *A* was the absorbance, MW was molecular weight of anthocyanin rounding 449.2 g/mol, DF was the dilution factor, *ε* was the molar absorptivity (26900), and *d* was the cuvette length (1 cm). Absorbance was calculated using [25]

(19) $$\begin{array}{c} A={\left(\;A_{520}-A_{700}\;\right)}_{pH1}-{\left(\;A_{520}-A_{700}\;\right)}_{pH4.5}. \end{array}$$

### 2.10. Kinetics of Antioxidant Activity and Colour Degradation

Kinetics of antioxidant activity and colour degradations were assumed to follow the first-order reaction. First-order reaction approach has been successfully used in describing the colour degradation of food products [24]. Since the antioxidant activity was linearly correlated to anthocyanins content, then the antioxidant activity degradation should obey the following equation:

(20) $$\begin{array}{c} \ln\left(\;\frac{I_{t}}{I_{0}}\;\right)=-k_{a}t. \end{array}$$

Similarly, the colour degradation can be described by

(21) $$\begin{array}{c} \ln\left(\;\frac{{TCR}_{t}}{{TCR}_{0}}\;\right)=-k_{c}t. \end{array}$$

*k*_*a*_ and *k*_*c*_ were the constant rate of antioxidant activity and colour degradations (minute^−1^). *I*_0_ and *I*_*t*_ were antioxidant activity of the sample at time 0 and *t*, while TCR_0_ and TCR_*t*_ were total colour ratio at time 0 and *t*. All the constants were obtained numerically with the help of Excel solver.

## 3. Results and Discussion

### 3.1. Effect of the Presence of Foaming Agent

**Figure 2:**
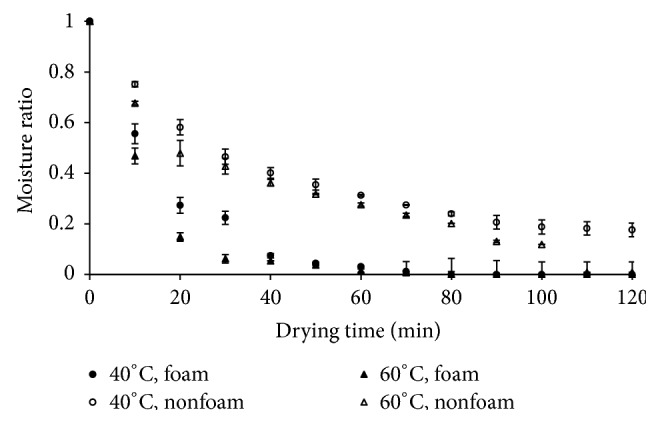
Moisture ratio of foam and nonfoam roselle extract drying at different air temperatures.

**Figure 3:**
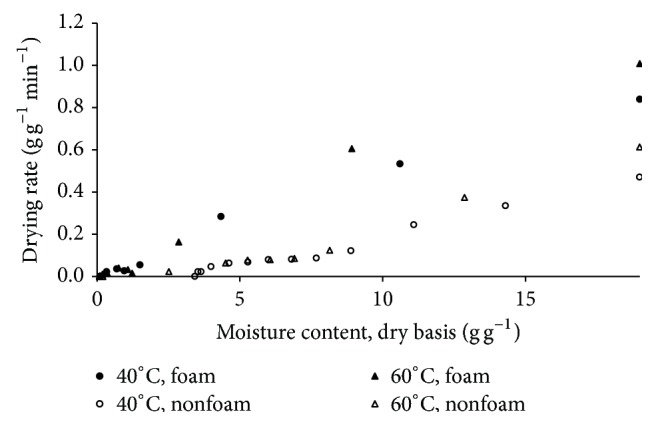
Drying rate of foam and nonfoam roselle extract drying at different air temperatures.

The roselle extract was dried with foaming agent and without foaming agent (nonfoam drying) at 40°C and 60°C. This step was expected to obtain dried product with moisture content of 0.11 gram of moisture per gram of dried product. The drying curves in the form of moisture ratio versus time were depicted in Figure 2. In all cases, the foaming agent played important role in speeding up water evaporation. As seen in Figure 2, the moisture ratio in product with foam reduced drastically. As expected, the foam drying had faster moisture evaporation leading to shorter drying time than that of nonfoam drying. During foam drying, the total surface area that was necessary for mass and heat transfer increased drastically as the honeycomb structure was formed through the destruction of the surface area of product into a plenty of smaller units. With this larger surface area, the moisture evaporation rate increased significantly [13]. Even, the drying rates of roselle extract with foam was 3 times higher than that of without foam (see Figure 3). The results were more significant compared with foam mat drying applied to the other food products using foam. Rajkumar et al. found a reduction of about 35 minutes to 40 minutes drying time during foam drying of mango pulp [21]. Meanwhile, Kandasamy et al. spent a half of drying time usually required for nonfoam drying of papaya pulp [15]. With shorter drying time or higher drying rate, foam mat drying offered a promising potential to save energy consumption.

### 3.2. Effect of Air Temperature and Relative Humidity

Table 1 presented the fact that the use of higher temperature shortened the drying time of roselle extract without foam. Similarly, Table 2 also showed that the drying time was shorter when higher temperatures were applied on foam drying. This behaviour can be explained by the increased drying rate caused by larger temperature gradient between drying air and foam which promote faster water evaporation rate [13, 15, 21].

Tables 1 and 2 showed that, at all temperatures studied, the use of low air relative humidity significantly reduced the drying time of both foam and nonfoam drying. With low air relative humidity, the driving force for moisture transfer was larger due to the lower equilibrium moisture content of the product. Hence, the roselle extract can be dried to even lower moisture content. Djaeni et al. reported that the use of low air relative humidity reduced the drying time of carrageenan significantly, especially for drying at low or medium temperatures (40 to 60°C) [26].

The drying rate constants were estimated using Newton model (see (5) and (6)), and the results were tabulated in Table 3. The model agreed well with the experimental data as indicated by high values of *R*^2^. This simple empirical model was chosen due to its simplicity and versatility for application in various drying experiments with different materials [21, 22]. The higher drying rate constants were achieved at higher temperature for both foam and nonfoam dryings. Ambekar et al. observed that the drying rate constant at 70°C was 2 times higher than at 50°C during foam drying of passion fruit pulp [27].

Arrhenius like model was used to describe the correlation between the drying rate constant (*k*_*d*_) and temperatures (see (7)). The value of Arrhenius constants were listed in Table 3. Using these constants, the drying rate and drying time for roselle extract at the desired drying temperatures can be well predicted. Based on the mass and heat convective transfer coefficients shown in Table 4, the Sherwood (Sh) number confirmed that mass convective flow was superior over the moisture diffusion and that moisture diffusion coefficients were significantly depending on the air temperature. Since the foam had a high porosity, it was expected that the internal motion of moisture was slower than its evaporation into the flowing air. Further, the Nusselt number (Nu) also confirmed that the convective heat transfer was faster than thermal diffusion because the high porosity of the food might hinder the heat conduction. Similar result was reported by Franco et al. in foam drying of yacon juice [18].

### 3.3. Antioxidant Activity Degradation

**Figure 4:**
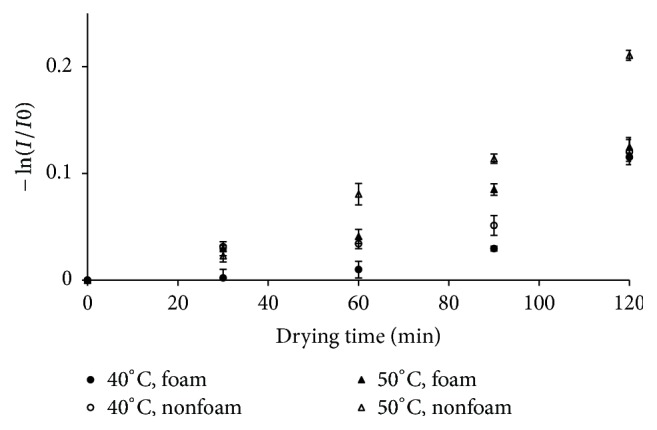
The antioxidant activity degradation at various drying conditions.

In this work, the antioxidant activity of roselle extract was expressed as percentage of inhibition (*I*%) to DPPH solution [23]. Degradation of antioxidant activity of dried roselle extract obtained from foam and nonfoam dryings at different air temperature is depicted in Figure 4. The dried roselle extracts experienced significant reduction of percentage of inhibition (*I*%) at both higher air temperature and longer drying time for all drying methods. In general, the antioxidant activity of roselle extracts obtained from foam drying was slightly higher than that of nonfoam drying indicating their higher total antioxidant content. Since the antioxidant activity of dried roselle extracts was mainly contributed by anthocyanins, which were sensitive to heat, then lower percentage of inhibition (*I*%) of dried roselle extract might be due to its lower anthocyanins content [6].

The antioxidant degradation rate constants *k*_*a*_ at various temperatures were calculated using (20), while their dependence on temperature was estimated using Arrhenius model (analog with (7)). The results were presented in Table 5. The higher value of *k*_*o*_ indicates that antioxidant degradation rate for nonfoam drying was faster than that of foam drying.

### 3.4. Colour Degradation

The dried roselle extracts obtained from nonfoam drying were all darker than those obtained from foam drying at the same drying conditions. With longer drying time, the sugar contained in the roselle extract may experience caramelisation and lead to giving browning effect on nonfoam drying products [28]. The stable foam available along the foam drying promoted faster moisture evaporation rate and resulted in shorter drying time, by which the product quality can be maintained [29].

**Figure 5:**
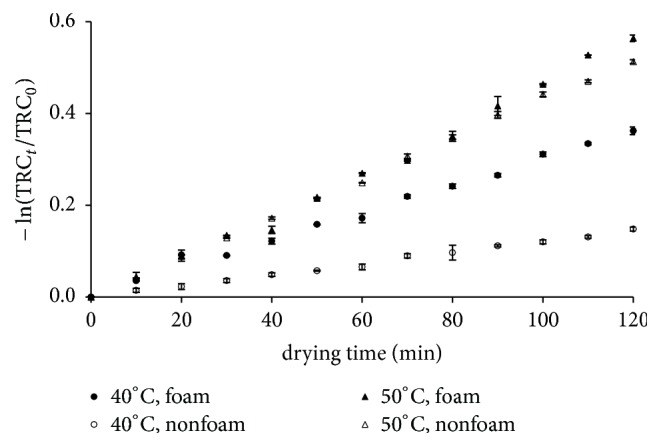
The colour degradation at various drying conditions.

Colour degradation in the form or TCR_*t*_/TCR_0_ was observed during the drying process as seen in Figure 5. Increase in drying temperatures led to reduction in the colour quality of the roselle extract during the drying process as indicated by darker colour. These phenomena can be explained by the formation of brown pigment during drying. In addition, the browning process also increased with an increase in drying temperature [30]. Reyes and Cisneros-Zevallos also reported that the degradation rate constant of colour of anthocyanins increased with temperatures as can be seen in the case of aqueous extracts of purple and red-flesh potatoes. The constant rate of the colour degradation at 50°C was 9.5 times higher than at 25°C [31].

During the drying process, anthocyanin content in roselle extract decreased with the increase of temperature (Table 6). In this case, by increasing drying temperatures 10°C the anthocyanin content can decrease about 15% (58.71 ± 1.52 mg/100 gram at 40°C to 46.85 ± 1.25 mg/100 gram at 50°C). The decrease of anthocyanin content caused the colour to change significantly as formulated by Cao et al. [32].

The colour degradation rate constants *k*_*c*_ at various temperatures were calculated using (21), while their dependence on temperature was estimated using Arrhenius like correlation (analog with (7)). The Arrhenius-like constants obtained in this study were listed in Table 7. It was clear that the colour degradation was faster for foam drying due to dual degradation effect by caramelisation and Maillard reactions. Addition of ovalbumin as foaming agent, which was rich in amino acids in its protein, led to the occurrence of Maillard reaction.

## 4. Conclusion

Roselle extract drying has been successfully performed with ovalbumin and glycerol mono stearate as foaming agent and foam stabiliser at various drying air conditions. The experiment results showed that the drying time of roselle extract with foaming agent was three times shorter than that of conventional drying without foam. Furthermore, drying time became shorter at higher air temperature and lower air relative humidity. With this condition, the antioxidant activity and colour can be well retained. The mathematical models have been also developed to represent the foam drying kinetics as well as antioxidant and colour degradation kinetics of the roselle extract. These model parameters were validated by experimental data. Therefore, the models can be used to estimate drying time for different drying temperature.

## Figures and Tables

**Table 1:** Predicted drying time of roselle extract without foam at various air conditions.

Aw/RH	Drying time (minutes)					
	30°C	40°C	50°C	60°C	70°C	80°C
0.0	572.6	410.4	256.8	214.9	153.6	116.9
0.2	587.2	419.7	261.9	218.4	155.4	117.7
0.4	656.0	451.7	275.9	226.0	158.5	118.8
0.6	nd^*∗*^	nd^*∗*^	377.0	247.2	164.5	120.5
0.8	nd^*∗*^	nd^*∗*^	nd^*∗*^	nd^*∗*^	178.6	123.2

nd^*∗*^: not dried (equilibrium moisture of the product was upper 10% wet basis).

**Table 2:** Predicted drying time of roselle extract with foam at various air conditions.

Aw/RH	Drying time (minutes)					
	30°C	40°C	50°C	60°C	70°C	80°C
0.0	163.0	131.6	93.3	84.9	66.9	55.5
0.2	167.6	134.9	95.4	86.5	67.8	56.0
0.4	184.6	145.3	101.0	90.0	69.7	56.9
0.6	nd^*∗*^	nd^*∗*^	187.7	101.5	73.9	58.8
0.8	nd^*∗*^	nd^*∗*^	nd^*∗*^	nd^*∗*^	99.1	63.6

nd^*∗*^: not dried (equilibrium moisture of the product was upper 10% wet basis).

**Table 3:** The constants of roselle extract drying at different temperature.

*T* (°C)	Nonfoam					Foam				
	*k*_*d*_ (min^−1^)	*D*_if_ × 10^9^ (m^2^ min^−1^)	*R* ^2^	*k*_*o*_ × 10^−2^	Ea (kJ mol^−1^)	*k*_*d*_ (min^−1^)	*D*_if_ × 10^9^ (m^2^ min^−1^)	*R* ^2^	*k*_*o*_ × 10^−2^	Ea (kJ mol^−1^)
40	0.017	3.13	0.90	2.38	29.77	0.052	8.56	0.92	3.12	19.92
50	0.026	4.28	0.86			0.071	11.69	0.93		
60	0.030	4.94	0.87			0.076	12.51	0.87		

**Table 4:** Heat and mass transfer coefficients.

*T* (°C)	*D*_wa_ × 10^−5^ (m^2^s^−1^)	Re^*∗*^	Sc^*∗*^	Sh^*∗*^	*k* (W m^−1^K^−1^)	Nu^*∗*^	*h* (W m^−1^K^−1^)	*h*_*m*_ × 10^−4^ (ms^−1^)
40	2.77	25.63	113483.61	2.81	27.15	3.01	408.05	3.903
50	2.96	24.62	83114.76	2.70	28.80	2.94	409.19	3.996
60	3.15	22.99	77646.68	2.55	28.59	2.84	406.49	4.028

Re^*∗*^: Reynold number, Sc^*∗*^: Schmidt number, Sh^*∗*^: Sherwood number, and Nu^*∗*^: Nusselt number.

**Table 5:** The Arrhenius parameters for antioxidant activity degradation.

*T* (°C)	Nonfoam				Foam			
	*k*_*a*_ (min^−1^)	*R* ^2^	*k*_*o*_ × 10^6^	Ea (kJ mol^−1^)	*k*_*a*_ (min^−1^)	*R* ^2^	*k*_*o*_ × 10^1^	Ea (kJ mol^−1^)
40	0.00094	0.85	5.9	58.42	0.0009	0.93	3.1	27.34
50	0.0025	0.94			0.001	0.97		
60	0.0036	0.90			0.0017	0.89		

**Table 6:** The total Anthocyanin content (TAC) of roselle extract drying with foam.

Variable	TAC (mg/L)
Mixture of roselle extract with foaming agent	83.49 ± 3.34
Dried product, 40°C	58.71 ± 1.52
Dried product, 50°C	46.85 ± 1.25
Dried product, 60°C	41.76 ± 0.47

**Table 7:** The Arrhenius parameters for colour degradation.

*T* (°C)	Nonfoam				Foam			
	*k*_*c*_ (min^−1^)	*R* ^2^	*k*_*o*_ × 10^6^	Ea (kJ mol^−1^)	*k*_*c*_ (min^−1^)	*R* ^2^	*k*_*o*_ × 10^2^	Ea (kJ mol^−1^)
40	0.0012	0.82	2.4	55.55	0.0030	0.92	4.4	30.86
50	0.0030	0.86			0.0048	0.93		
60	0.0043	0.87			0.0061	0.87		
